# Supplementary material for: Assessing motivators for utilizing family planning services among youth students in higher learning institutions in Dodoma, Tanzania: Protocol for analytical cross sectional study
Source: PLoS One. 2023 Mar 10;18(3):e0282249. doi: 10.1371/journal.pone.0282249 (PMC10004694; doi:10.1371/journal.pone.0282249)
Supplement: S1 File — (PDF) [file pone.0282249.s001.pdf]

THE UNIVERSITY OF DODOMA
OFFICE OF THE DEPUTY VICE CHANCELLOR-ARC
DIRECTORATE OF RESEARCH, PUBLICATIONS AND CONSULTANCY
P.O. Box 259
DODOMA, TANZANIA
TEL: +255-026-2310002 FAX: +255-026-2310012
EMAIL: dvcarc@udom.ac.tz;
Website address:www.udom.ac.tz

Ref. No. MA.84/261/02/ 18th March, 2022

To: Ms. Upendo Munuo
The University of Dodoma

RE: REQUEST FOR ETHICAL CLEARANCE

This is to inform you that the proposal titled **"Assessing the Motivators and Demotivates for Utilizing Family Planning Services among Higher Learning Institution Youth Students: An Analytical Cross- Sectional Study in Dodoma Region."** has been granted ethical clearance.

Furthermore, as the Principal Investigator of the study, the following conditions must be fulfilled:

- Progress report is submitted to the University of Dodoma.
- Permission to publish the results is obtained from the University of Dodoma.
- Copies of final publications are made available to the University of Dodoma.
- Sites: **Dodoma Region.**

Best regards,

Dr. Alex Mongi
For: Chairperson - Institutional Research Review Committee (IRREC)

C: C: Deputy Vice Chancellor-Academic, Research and Consultancy
