# Supplementary material for: Assessing motivators for utilizing family planning services among youth students in higher learning institutions in Dodoma, Tanzania: Protocol for analytical cross sectional study
Source: PLoS One. 2023 Mar 10;18(3):e0282249. doi: 10.1371/journal.pone.0282249 (PMC10004694; doi:10.1371/journal.pone.0282249)
Supplement: S1 Questionnaire — (DOCX) [file pone.0282249.s002.docx]

Questionnaire

Dear participant, you are humbly requested to participate in this study which aims to assess **motivators for utilizing family planning services among** youth students in higher learning institutions in Dodoma region. The result of this study will be used to enlighten policy and programme decisions in the learning institutions and also add to the existing stock of knowledge in this study area to increase the family planning service utilization. I would be grateful if you could provide me with accurate and detailed information. Your participation is highly valued and appreciated. I assure you that all your comments will remain confidential.

**Date**: ………….. **Name of Study Site:** ……………………………………………..

**Respondent code:**…….................. **Questionnaire number**……………………….

**PART I: DEMOGRAPHIC INFORMATION OF PARTICIPANTS**

1. How old are you? **Specify**................. (in years)
2. What is your sex? Please **encircle** one
3. Female
4. Male
5. What is your level of education? Please **encircle** that is applicable.
6. Certificate
7. Diploma
8. Undergraduate
9. Which year of the study are you?
10. First
11. Second
12. Third
13. Which study programme are you currently enrolled?
14. Health related
15. Non-health related
16. What is the name of the institution you are currently enrolled for study?
17. UDOM
18. SJUT
19. MIPANGO
20. CBE
21. What is your home residence?
22. In campus
23. Off campus
24. What is your marital status?
25. Single
26. Cohabited
27. What is your religion among the following?
28. Christian: Specify your denomination……………….
29. Muslim
30. Others: Specify ………………………………….
31. Does your family have a habit of discussing the FP issues with you?
32. Yes
33. No
34. At home who are you living with? Please **encircle** one.
35. Both Parents
36. Single parent
37. Other relatives

**PART II: QUESTIONS ASSESSING PERSONAL REPRODUCTIVE CHARACTERISTICS**

| **QNO** | **QUESTIONS** | **RESPONSES** |
| --- | --- | --- |
| 1 | How many sexual partner (s) have you had in the past three years? | ………………………………. |
| 2 | Have you ever had sexual intercourse? | (a)YES  (b)NO |
| 3 | Have you had sexual intercourse in the past 12 months? | (a)YES  (b)NO |
| 4 | If **YES,** with how many partners have you had sexual intercourse in the past 12 months? | ……………………….. |
| 5 | Have you discussed about FP methods with your sexual partner within the past 12 months? | (a)YES  (b)NO |
| 6 | Have you ever exposed to mass media to access information and education on at least two FP services listed below within the past 12 months? Condom, Abstinence, Contraception, Counselling | (a)YES  (b)NO |
| 7 | If Yes, which mass media did you expose to?  **If no skip question number 7** | (a)Radio  (b) Television  (c) Magazine  (d) Newspaper  (e). Pamphlet  (f) Other specify___________ |
| 8 | Which of the following do you think influence utilisation of FP services? | (a) Parents decision  (b) Partner  (c) Religious beliefs  (d) Influence from friends  (d) Cultural beliefs  (e) Cost of the FP services  (f) Other specify…………. |
| 9 | At which age did you have your first sexual intercourse? | < 15 years  15-19  20-24 |

**Part III: QUESTIONS REGARDING PERCEPTIONS ON USE OF CONTRACEPTIVES**

**Instructions**: Please read the following statements and rate how much you personally agree or disagree with each statement by putting a tick **(√)** in an appropriate column that correspond to your feeling.

| *KEY* | *Strongly Agree* | *Agree* | *Neither Agree Nor Disagree* | *Disagree* | *Strongly Disagree* |
| --- | --- | --- | --- | --- | --- |
|  | *1* | *2* | *3* | *4* | *5* |

| **SN** | **ITEM** | **5** | **4** | **3** | **2** | **1** |
| --- | --- | --- | --- | --- | --- | --- |
| 1 | FP uses are meant only for married persons |  |  |  |  |  |
| 2 | FP methods are expensive |  |  |  |  |  |
| 3 | FP use leads to infertility |  |  |  |  |  |
| 4 | The process of getting the preferred FP method is often embarrassing |  |  |  |  |  |
| 5 | FP method should not be used; they are bad |  |  |  |  |  |
| 6 | Contraceptives have significant side effects |  |  |  |  |  |
| 7 | Religious beliefs are a barrier to using FP services among the youths |  |  |  |  |  |
| 8 | FP commodities are always not available at the facility |  |  |  |  |  |
| 9 | Waiting time hinders me to use FP services |  |  |  |  |  |
| 10 | Service provider refuse to give me the service |  |  |  |  |  |
| 11 | The services are not friendly to the youths |  |  |  |  |  |
| 12 | Healthcare providers are harsh to the youths/adolescents |  |  |  |  |  |
| 13 | There is no reproductive health clinic providing FP services to the youths |  |  |  |  |  |
| 14 | Class periods are tight that no time to visit the clinic for FP services |  |  |  |  |  |
| 15 | FP services use does not prevents unsafe abortions and preventable maternal deaths |  |  |  |  |  |
| 16 | FP use is not a preventive measure of unintended pregnancies and sexually transmitted infection. |  |  |  |  |  |
| 17 | FP services use does not prevent the youths from human immunodeficiency virus infection and acquired immune deficiency syndrome (HIV/AIDS). |  |  |  |  |  |
| 18 | FP use doesn’t reduce the risks of uterine and fallopian cancer |  |  |  |  |  |
| 19 | FP use does not prevent symptomatic pelvic inflammatory disease. |  |  |  |  |  |
| 20 | FP services use can cause infertility |  |  |  |  |  |
| 21 | FP use can cause cervical cancer |  |  |  |  |  |
| 22 | Condom use may lead to hypersensitivity (allergy) |  |  |  |  |  |
| 23 | Condom use may slip into the uterus |  |  |  |  |  |
| 24 | Implants may move away from the site of insertion causing deaths |  |  |  |  |  |
| 25 | FP services use among the youths is not good for health |  |  |  |  |  |
| 26 | FP services use is associated with ever wetting of the vagina which indicates unhealthiness and may be defined negatively by the partner |  |  |  |  |  |
| 27 | Amenorrhea due to use of any FP method is not healthy for a woman, especially the youth |  |  |  |  |  |

**PART IV: QUESTIONS ABOUT KNOWLEDGE OF FAMILY PLANNING METHODS**

| **NO** | **ITEM** | **YES (√)** | **NO (X)** |
| --- | --- | --- | --- |
| 1 | Have you ever heard about FP services? |  |  |
| 2 | What is family planning? **(encircle the correct response)** |  |  |
|  | 1. Don’t know |  |  |
|  | 1. A measure to reduce child birth |  |  |
|  | 1. A measure to prevent unwanted pregnancy |  |  |
|  | 1. A measure to space child birth |  |  |
|  | 1. A measures to reduce number of children and unwanted pregnancy |  |  |
|  | 1. A measure to reduce unwanted pregnancy, space and reduce childbirth |  |  |
| 3 | Have you ever heard about modern contraceptive? |  |  |
| 4 | What are modern family planning methods? **(encircle the correct response)** |  |  |
|  | 1. Don’t know |  |  |
|  | 1. Measure to promote number of children |  |  |
|  | 1. Use of hormonal, artificial material or minor surgery to prevent unwanted pregnancy, space or limit birth |  |  |
|  | 1. Use of Bills ovulation methods to prevent unwanted pregnancy, space or limit birth |  |  |
| 5 | Have you ever heard about traditional contraceptives? |  |  |
| 6 | What are traditional family planning methods? **(encircle the correct response)** |  |  |
|  | 1. Don’t know |  |  |
|  | 1. Use of hormonal methods to prevent unwanted pregnancy, or space birth |  |  |
|  | 1. Use of artificial and barrier methods to prevent unwanted pregnancy, or space birth |  |  |
|  | 1. Methods of planning sexual intercourse by observing some signs occurring in the woman body in the fertile and infertile periods of the menstrual cycle |  |  |
|  | 1. Methods used to prevent sexually transmitted infection |  |  |
| 7 | Have you ever heard about emergency family planning? |  |  |
| 8 | What is emergency family planning? **(encircle the correct response)** |  |  |
|  | 1. Don’t know |  |  |
|  | 1. Use of barrier methods to prevent unwanted pregnancy, or space birth |  |  |
|  | 1. Use of hormonal methods to prevent pregnancy following unprotected sexual intercourse |  |  |
|  | 1. Methods used to prevent sexually transmitted infection |  |  |
| 9 | If YES in question 1, 3, 5 and 7, what was the Source of information? |  |  |
|  | 1. Friends/peers |  |  |
|  | 1. Health facility |  |  |
|  | 1. Pharmacy/shop |  |  |
|  | 1. Television |  |  |
|  | 1. Health care workers |  |  |
|  | 1. Radio |  |  |
|  | 1. Internet |  |  |
|  | 1. Family member |  |  |
|  | 1. Partner |  |  |
|  | 1. Poster |  |  |
|  | 1. Other specify……………… |  |  |
| 10 | Which among the following are FP methods ***(Tick all that apply)*** |  |  |
|  | 1. Condom |  |  |
|  | 1. Pills |  |  |
|  | 1. Injectable/Depo-Provera |  |  |
|  | 1. Implants |  |  |
|  | 1. Intrauterine devices |  |  |
|  | 1. Female sterilization (BTL) |  |  |
|  | 1. Vasectomy |  |  |
|  | 1. Diaphragm |  |  |
|  | 1. Spermicides |  |  |
|  | 1. Emergency contraception |  |  |
|  | 1. Hysterectomy |  |  |
|  | 1. Myomectomy |  |  |
| 11 | Which among the following are traditional FP methods? ***(Tick all that apply)*** |  |  |
|  | 1. Abstain |  |  |
|  | 1. Withdrawal |  |  |
|  | 1. Periodic abstinence |  |  |
|  | 1. Lactational amenorrhea |  |  |
|  | 1. Pills |  |  |
|  | 1. Implants |  |  |
|  | 1. Diaphragm |  |  |
| 12 | Can you get pregnancy when using withdrawal method? |  |  |
| 13 | Which among the following are modern FP methods? ***(Tick all that apply)*** |  |  |
|  | 1. Condom |  |  |
|  | 1. Pills |  |  |
|  | 1. Injectable/Depo-Provera |  |  |
|  | 1. Implants |  |  |
|  | 1. Intrauterine devices |  |  |
|  | 1. Female sterilization (BTL) |  |  |
|  | 1. Vasectomy |  |  |
|  | 1. Diaphragm |  |  |
|  | 1. Spermicides |  |  |
|  | 1. Emergency contraception |  |  |
|  | 1. Withdraw |  |  |
|  | 1. Calendar |  |  |
| 14 | Which among the following are emergency contraceptives FP methods? ***(Tick all that apply)*** |  |  |
|  | 1. Diaphragm |  |  |
|  | 1. Depo-Provera |  |  |
|  | 1. IUCD |  |  |
|  | 1. Levonorgestrel pills |  |  |
|  | 1. Combine oral contraceptive |  |  |
|  | 1. Withdraw |  |  |

**PART V: QUESTIONS ABOUT FAMILY PLANNING UTILISATION**

**Encircle the appropriate response for each question below**

1. Have you ever used any means to delay or avoid getting pregnancy in the last 12 months?
2. Yes
3. No
4. Have you ever used any modern contraceptive service in the last 12 months?
5. Yes
6. No
7. If **YES**, what type of method did you use?
8. Male condom
9. Pill
10. Injectable
11. Implant
12. IUCD
13. Female condom
14. P2 (emergency contraceptives)
15. Have you ever used emergency contraceptives?
16. Yes
17. No
18. Have you ever used traditional FP methods?
19. Yes
20. No
21. If **YES**, what method did you use?
22. Withdraw
23. Periodic abstinence
24. Lactational amenorrhea
25. Have you received any information and education service regarding FP services from health worker working in any of RH clinics delivery points in the past 12 months?
26. Yes
27. No
28. If **YES,** what type of information and education have you received?
29. Regarding sexual health
30. Regarding contraception
31. Regarding STIs and treatment
32. Regarding unsafe abortion

1. Do you always have access to FP services in your area?
2. Yes
3. No
4. Do you know where to get FP services by yourself when you need it?
5. Yes
6. No
7. Have you ever experienced any side effect(s) from any method of the FP methods ever used?
8. Yes
9. No
10. Do you intend to use FP methods in the future?
11. Yes
12. No

**PART VI: QUESTIONS REGARDING HEALTH SERVICES PROVISION ENVIRONMENT**

1. Are FP services available at your nearest health facility?
2. Yes
3. No
4. Have you discussed at least two methods of FP with your health workers within the past 12 months?
5. Yes
6. No
7. Are the FP services provided free of charge?
8. Yes
9. No
10. If no; can you afford to pay for FP methods?
11. Yes
12. No
13. Is the service provided at the nearest facility meet the following characteristics?
14. healthcare providers treat youth clients with respect
15. Yes
16. No
17. healthcare providers honour privacy and confidentiality of youth clients
18. Yes
19. No
20. healthcare providers allow adequate time for adolescent client and provide interaction and availability of peer counsellors at the facility
21. Yes
22. No
23. How can you estimate the distance from your place of residency to your nearest health facility?
24. Within 5 kms
25. 5–10 kms
26. Above 10 kms
27. How can you estimate the time consumed to wait for FP services from your nearest health facility?
28. 5- 9 min
29. 10-14 min
30. ≥15 min
31. Are adolescent-friendly services available at your nearest health facility?
32. Yes
33. No
