# Supplementary material for: Assessing motivators for utilizing family planning services among youth students in higher learning institutions in Dodoma, Tanzania: Protocol for analytical cross sectional study
Source: PLoS One. 2023 Mar 10;18(3):e0282249. doi: 10.1371/journal.pone.0282249 (PMC10004694; doi:10.1371/journal.pone.0282249)
Supplement: Attachment — Submitted filename: PONE-D-22-21995_reviewerfb.pdf [file pone.0282249.s003.pdf]

PLOS ONE

Assessing Motivators for Utilizing Family Planning Services among Youth Students in Higher Learning Institutions in Dodoma, Tanzania: Analytical Cross Sectional Study protocol

--Manuscript Draft--

**Manuscript Number:**	PONE-D-22-21995
**Article Type:**	Study Protocol
**Full Title:**	Assessing Motivators for Utilizing Family Planning Services among Youth Students in Higher Learning Institutions in Dodoma, Tanzania: Analytical Cross Sectional Study protocol
**Short Title:**	Assessing Motivators for Utilizing Family Planning among Youth Students in Higher Learning Institutions
**Corresponding Author:**	Upendo Munuo, Master<br>University of Dodoma College of Health and Allied Sciences: The University of Dodoma College of Health Sciences<br>Dodoma, TANZANIA, UNITED REPUBLIC OF
**Keywords:**	
**Abstract:**	Background<br>Contraceptive services utilization is an important intervention in averting the impact of unwanted and unplanned pregnancy among youth which is an obstacle to the higher learning institution youth students in attaining their educational goals. Therefore, the current protocol aims to assess the motivators for family planning services utilization among youth students in higher learning institution in Dodoma Tanzania.<br>Objective of the study<br>The broad objective is to assess motivators for utilizing family planning services among youth students in higher learning institutions in Dodoma, Tanzania.<br>Methods<br>This study will be a cross-sectional study with quantitative approach. A multistage sampling technique will be employed in studying 421 youth students aged between 18 to 24 years using structured interviewed-administered questionnaire adopted from the previous studied. At CI of 95% both descriptive and inferential statistics will be wined and percentages, frequencies, p-values, Crude Odds Ratio (COR) and Adjusted Odds Ratio (AOR) will be presented respectively. The study outcome will be family planning service utilization and independent variables will be services provision environment, knowledge factors, and perception factors.
**Order of Authors:**	Upendo Munuo, Master
	Fabiola Vincent Moshi, PhD
**Additional Information:**	
**Question**	**Response**
**Financial Disclosure**<br><br>Enter a financial disclosure statement that describes the sources of funding for the work included in this submission. Review the submission guidelines for detailed requirements. View published research articles from *PLOS ONE* for specific examples.<br><br>This statement is required for submission and **will appear in the published article** if	The authors received no specific funding for this work

Powered by Editorial Manager® and ProduXion Manager® from Aries Systems Corporation

the submission is accepted. Please make sure it is accurate.<br><br>**Unfunded studies**<br>Enter: *The author(s) received no specific funding for this work.*<br><br>**Funded studies**<br>Enter a statement with the following details:<br>• Initials of the authors who received each award<br>• Grant numbers awarded to each author<br>• The full name of each funder<br>• URL of each funder website<br>• Have or will the sponsors or funders play any role in the study design, data collection (when applicable) and analysis, decision to publish, or preparation of the manuscript?<br>• **NO** - Include this sentence at the end of your statement: *The funders had and will not have a role in study design, data collection and analysis, decision to publish, or preparation of the manuscript.*<br>• **YES** - Specify the role(s) played.<br><br>* typeset	
**Competing Interests**<br><br>Use the instructions below to enter a competing interest statement for this submission. On behalf of all authors, disclose any competing interests that could be perceived to bias this work—acknowledging all financial support and any other relevant financial or non-financial competing interests.<br><br>This statement is required for submission and **will appear in the published article** if the submission is accepted. Please make sure it is accurate and that any funding sources listed in your Funding Information later in the submission form are also declared in your Financial Disclosure statement.<br><br>View published research articles from *PLOS ONE* for specific examples.	The authors have declared that no competing interest exist.

Powered by Editorial Manager® and ProduXion Manager® from Aries Systems Corporation

**NO authors have competing interests**<br><br>Enter: *The authors have declared that no competing interests exist.*<br><br>**Authors with competing interests**<br><br>Enter competing interest details beginning with this statement:<br><br>*I have read the journal's policy and the authors of this manuscript have the following competing interests: [insert competing interests here]*<br><br>* typeset	
**Ethics Statement**<br><br>Enter an ethics statement for this submission. This statement is required if the study involved:<br><br>• Human participants<br>• Human specimens or tissue<br>• Vertebrate animals or cephalopods<br>• Vertebrate embryos or tissues<br>• Field research<br><br>Write "N/A" if the submission does not require an ethics statement.<br><br>General guidance is provided below. Consult the submission guidelines for detailed instructions. **Make sure that all information entered here is included in the Methods section of the manuscript.**	University of Dodoma Research Ethical Committee board, approval No. Ref. No. MA.84/261/02/'A'/91.<br>Both written and oral consent will be obtained from study participants

Powered by Editorial Manager® and ProduXion Manager® from Aries Systems Corporation

**Format for specific study types**<br><br>**Human Subject Research (involving human participants and/or tissue)**<br>• Give the name of the institutional review board or ethics committee that approved the study<br>• Include the approval number and/or a statement indicating approval of this research<br>• Indicate the form of consent obtained (written/oral) or the reason that consent was not obtained (e.g. the data were analyzed anonymously)<br><br>**Animal Research (involving vertebrate animals, embryos or tissues)**<br>• Provide the name of the Institutional Animal Care and Use Committee (IACUC) or other relevant ethics board that reviewed the study protocol, and indicate whether they approved this research or granted a formal waiver of ethical approval<br>• Include an approval number if one was obtained<br>• If the study involved *non-human primates*, add *additional details* about animal welfare and steps taken to ameliorate suffering<br>• If anesthesia, euthanasia, or any kind of animal sacrifice is part of the study, include briefly which substances and/or methods were applied<br><br>**Field Research**<br><br>Include the following details if this study involves the collection of plant, animal, or other materials from a natural setting:<br>• Field permit number<br>• Name of the institution or relevant body that granted permission	
**Data Availability**<br><br>Authors are required to make the data underlying their research findings fully available, without restriction.<br><br>If the manuscript reports pilot or preliminary results, the data underlying those results must be made available at the time of publication. A Data Availability	N/A - the protocol does not report results

Powered by Editorial Manager® and ProduXion Manager® from Aries Systems Corporation

Statement describing where the data can be found is required at submission. Your answers to this question constitute the Data Availability Statement and will be published in the article, if accepted. When legal or ethical requirements prohibit public data sharing, authors must indicate how researchers may obtain access to the data. See the PLOS Data Policy and FAQ for detailed information.<br><br>**Important:** Stating 'data available on request from the author' is not sufficient. If your data are only available upon request, select 'No' for the first question and explain your situation in the text box.<br><br>For protocols without pilot or preliminary data, authors are strongly encouraged to state how they plan to share research data from their study when it is completed or published.<br><br>Do the authors confirm that EITHER the protocol does not report results OR all data underlying the preliminary results will be made fully available without restriction?	
**Describe where the data may be found in full sentences. If you are copying our sample text, replace any instances of XXX with the appropriate details.**<br><br>• If **no pilot data are reported** in the manuscript, please enter the following: *No datasets were generated or analysed during the current study. All relevant data from this study will be made available upon study completion.*<br>• If the manuscript reports pilot data **held in a public repository**, include URLs, accession numbers or DOIs. If this information will only be available after acceptance, indicate this by ticking the box below. For example: *All XXX files are available from the XXX database*	Deidentified research data will be made publicly available when the study is completed and published.

Powered by Editorial Manager® and ProduXion Manager® from Aries Systems Corporation

(accession number(s) XXX, XXX.).<br>• If the manuscript reports pilot data contained **within the manuscript and/or Supporting Information files**, enter the following:<br>*All relevant data are within the manuscript and its Supporting Information files.*<br>• If neither of these applies but you are able to provide **details of access elsewhere**, with or without limitations, please do so. For example:<br><br>*Data cannot be shared publicly because of [XXX]. Data are available from the XXX Institutional Data Access / Ethics Committee (contact via XXX) for researchers who meet the criteria for access to confidential data.*<br><br>*The data underlying the results presented in the study are available from (include the name of the third party and contact information or URL).*<br>• This text is appropriate if the data are owned by a third party and authors do not have permission to share the data.<br><br>For study protocols with data management or sharing plans, authors are encouraged to briefly describe in their Data Availability Statement how data they generate will be made accessible when the study is completed. For example:<br>• Deidentified research data will be made publicly available when the study is completed and published.<br><br>* typeset	
Additional data availability information:	Tick here if the URLs/accession numbers/DOIs will be available only after acceptance of the manuscript for publication so that we can ensure their inclusion before publication.; Tick here if your circumstances are not covered by the questions above and you need the journal’s help to make your data available.

Manuscript

Click here to access/download;Manuscript;STUDY PROTOCOL AUGUST 5 FOR SUBMISSION.docx

Assessing Motivators for Utilizing Family Planning Services among Youth Students in Higher Learning Institutions in Dodoma, Tanzania: Protocol for Analytical Cross Sectional Study

Authors: Upendo Munuo[1*] & Fabiola Vincent Moshi[2]

Authors' affiliation:

[1]Department of Clinical Nursing, School of Nursing and Public Health, the University of Dodoma P.O. Box 254, Dodoma Tanzania, [2]Department of Nursing Management and Education, School of Nursing and Public Health, the University of Dodoma P.O. Box 254, Dodoma Tanzania

***Corresponding author**

Email addresses

Upendo Munuo: pendomunuo@yahoo.com

Fabiola Vincent Moshi: fabiola.moshi@gmail.com

Abstract

Background

Contraceptive services utilization is an important intervention in averting the impact of unwanted and unplanned pregnancy among youth which is an obstacle to the higher learning institution youth students in attaining their educational goals. Therefore, the current protocol aims to assess the motivators for family planning services utilization among youth students in higher learning institution in Dodoma Tanzania.

Tautology

Objective of the study

The broad objective is to assess motivators for utilizing family planning services among youth students in higher learning institutions in Dodoma, Tanzania.

Methods

This study will be a cross-sectional study with quantitative approach. A multistage sampling technique will be employed in studying 421 youth students aged between 18 to 24 years using structured interviewed-administered questionnaire adopted from the previous studied. At CI of 95% both descriptive and inferential statistics will be wined and percentages, frequencies, p-values, Crude Odds Ratio (COR) and Adjusted Odds Ratio (AOR) will be presented respectively. The study outcome will be family planning service utilization and independent variables will be services provision environment, knowledge factors, and perception factors.

Not certain how these will be analysed. How will confounding factors be controlled and analysed

Introduction

World Health Organization (WHO) defines youth as the phase an individual is no longer a child but is not yet an adult. Age wise, adolescent is regarded from 10 to 24 years [1]. Globally, 1.2 billion out of the estimated 3 billion young population aged less than 25 years [2]. In sub-Sahara Africa, nearly 1 in every 3 individuals is aged between 10 to 24 years [3]. Young girls are the most vulnerable group for not utilizing Sexual Reproductive Services including family planning, yet they are given little attention, especially in sub-Saharan Africa. This is due to the fact that they are victims of early marriage, teenage pregnancy, unwanted and unplanned pregnancy, and sexual transmitted infections associated with early debut to unsafe sexual behaviors [4].

In low and middle income countries like Tanzania one-third of the women were wedded at the age below 18 years, while one in nine are married at 15 years old [5]. It is estimated that 11% of births occur among adolescents worldwide whereby more than 90% of these births occur in low and middle income countries [6]. Sub-Saharan Africa is among of the region with highest teenage pregnancy rates and yet with the lowest rates of family planning (FP) uptakes [7].

Tanzania, in particular, adolescents comprise almost one quarter (24%, or 12.8 million) of the total population [8]. The issue of health services provision such as reproductive health, nutrition services and screening services to young people is a concern which requires attention from government and different stakeholders. Utilization of sexual and reproductive health services (SRHs) including FP services among adolescents is a crucial strategy in averting teenage pregnancies, unwanted and unplanned pregnancy, unprotected sexual intercourse which can results to reduction of maternal morbidity and mortality and STIs risks [7].

Increasing utilisation

Promoting FP services use among adolescents is vital in achieving universal access to sexual and reproductive health services [9]. Sub-Sahara Africa (SSA) have the greatest unmet need for family planning use [10], and young people reported to under-utilize FP services compared to other groups of women of the reproductive age in the region [7]. In connection to that, International Center for Research on Women (ICRW) analysis report that unmet need for family planning use is greater among unmarried adolescents than those who are married, though married adolescents aged 15-19 experience a higher proportion of unmet need compared to all married women [5].

Regarding the report of International Conference for Population and Development (ICPD), user-friendly sexual and reproductive health (SRH) services to adolescents is a paramount

agenda [11]. User-friendly SRH services offers adolescents with the opportunity to get accurate reproductive health information, education, counseling and health promotion activities [12]. In addition adolescent regard SRH services user-friendly if the particular service maintains respect and confidentiality [1]. According to WHO guideline entitled “Making Health Services Adolescent Friendly” SRH/FP services targeted for them should bear the quality elements which include accessibility, acceptability, equitable, appropriate, and effective [1]. Adolescents need to be offered FP services at the environment which favor their youth rights and feeling welcomed by health care providers [13].

Family planning methods if consistently and correctly used will prevent unplanned or unintended pregnancies and thus curtailing preventable maternal morbidity and mortality amongst adolescent girls [14]. It is predicted that universal access and utilization of family planning methods amongst adolescents could lead to a decrease of 2.1 million unplanned and or unintended births, 3.2 million unsafe abortions, and 5600 maternal deaths each year [15]. Utilization of family planning methods further provides adolescents with an opportunity to make informed decisions about when they want to have their children [16].

Despite of these benefits, young people face a numerous issues which are hindrance to their utilization of FP services. Young people reported receiving FP services with lower content to the service they received from healthcare providers elsewhere in SSA [7]. According to 2015/16 TDHS many young people were reported to have a limited education regarding SRH services including FP methods a result, they continued to engage in risky sexual practices and behaviors such as multiple and concurrent partnerships and inconsistent condom use [17].

Yet teen pregnancy remains a challenge to young people in schools, colleges and community as this may be contributed by either inadequate utilization of FP services which is considered an imperative strategy towards reducing preventable teen pregnancies, and other related complications [7,13]. Therefore, the broad objective of this study is to assess the motivators Determinants for family planning services utilization of among higher learning institution youth students in Dodoma Tanzania. To achieve this broad objective, the specific objectives are:

1. To determine the prevalence of FP services utilization among higher learning youth students in Dodoma Tanzania.
2. To determine level of knowledge of FP services among higher learning youth students in Dodoma Tanzania.

3. To determine perception of FP services utilization among higher learning youth students in Dodoma Tanzania.
4. To assess the FP provision environment among higher learning youth student in Dodoma Tanzania.
5. To assess the motivators for FP services utilization among higher learning youth students in Dodoma Tanzania.

This knowledge gap is not sufficiently demonstrated

The findings from this study are expected, to bridge knowledge gap by addressing motivators for family planning utilization among youth students in higher learning institution in Dodoma Tanzania, which will help to improve the utilization of family planning services. Moreover, the findings will help to develop different strategies, which will increase utilization of family planning services among youth students from higher learning institution in Dodoma Tanzania. Furthermore, the findings will act as catalyst for other researchers to conduct more in-depth research on the utilization of family planning services among youth students in higher learning institution in Dodoma Tanzania and elsewhere in the world.

Methods

Study area

The study will be conducted in Dodoma City. Dodoma city has the size of 2769 square kilometers. It has: 453,844 households, 41 wards 170 streets 42 villages and 89 hamlets. The Dodoma city is located at the south eastern end of the Tanzania Central Plateau at an elevation of 1,200 meters above sea level. The town is located at the geographical center of the country on the vital Central Railway line; and on major crossroad of the National East West trunk road and the famous north to south Cape to Gairo Great North Road, which passes in Tanzania through Mbeya, Iringa, Dodoma, Babati and Arusha. The town is 465 kilometers from Dar-es-Salaam, and 425 kilometers from Arusha situated at 6oo south of the Equator. It has the total population of more than 2.6 million with growth rate of 3.1% [18]. The total fertility rate in Dodoma city is 5.7% [19].

Study design

This will be Analytical cross sectional study design underpinned with quantitative approach assess the motivators for family planning services utilization of among higher learning institution youth students in Dodoma region, Tanzania

Study population

No information on why four institutions will be selected. If there are only eight institutions, why not include a representative sample from all

The study population will be all youth students from selected higher learning institutions of Dodoma city and youth students aged between 18-24 years in the first to final year at the selected institutions will be included in the study. All youth students (aged 18–24 years) who will be married, sick or well but unwilling to participate in the study will be excluded from this study.

Sample size calculation and sampling technique

The Cochran's formula [20] will be used to determine the minimum sample size required for this study.

$$n = \frac{z^2 P(100 - P)}{d^2} = \frac{1.96^2 \times 47(100 - 47)}{5^2} = 383$$

Where:

- n=desired sample size.
- z= critical value at 95% confidence level corresponds to 1.96
- d =marginal error (desired level of precision which is 5%).
- P= prevalence of FP use among youth students (47%) [21].

To cater for potential attrition (unresponsiveness) 10% of the minimum sample size will be added up as shown below:

- $10/100 \times 383 = 38.3 \approx 38 + 383 = 421$

Therefore, the total sample size for this study will be 421 participants.

Why? How will this be provided for in the analysis?

A multi-stage sampling will be employed to select participants from higher learning institutions in which two stages were involved.

- **At the first stage,** four out of eight higher learning institutions available in Dodoma city will be selected by simple random sampling technique.

- **Second stage**: After selection of four institutions, a proportionate stratified sampling technique will be employed to determine the sample size from each selected institution to give a total of 421 participants. This will worked out following this approach: (i) determined the number of eligible youth students from each institution; (ii) employed the formula below to calculate representative sample size from each selected institution. (iii) a

systematic sampling technique was used to obtain the samples from each selected institution

$$n_i = \left(\frac{n}{N}\right) N_i$$

Where:

n = total sample size to be selected, N = total population, N_i = total population of each facility, and n_i =sample size from each facility.

Data collection tools and methods

Questionnaire not attached

A structured questionnaire adopted from the previously studies and thereafter modified accordingly to fit the current study will be used to collect the relevant data [22,23]. The questionnaire comprised of six parts: (i) demographic profiles, (ii) individual sexual characteristics, (iii) perception on FP use, (iv) knowledge on FP services, (v) FP services utilization, and (vi) Health provision environment for FP services. The questionnaire will be constructed in English comprising both open and closed questions.

Reported as interviewer administered

Self-administered questionnaire will be employed to collect relevant data from the selected youth. Two pre-trained assistant data collectors will be involved in the data collection procedure. The research assistance will be nurse practitioner. The procedure will include self-introduction to the youth, introduction of the topic of study and the purpose of the study. Then, the researchers requested their participation in the study. The questions on the data collection tool will be organized in such a way that sensitive questions will be asked later. The participants will be assured of free participation and withdrawal from the study at any time.

Definition and measurement of variables

i. **Dependent variables (Family planning utilization).** The dependent variable for this study will be Family planning services utilization. Family planning utilization will be define as the participant who will report ever used of any type of family planning methods in the past 12 months and be able to mention it.

ii. **Independent variables:** The independent variables for this study will be; socio-demographic and sexual characteristics, family planning knowledge, perception towards family planning utilization and family planning services provision environment.

Data processing and analysis

Collected data will be cleaned and analyzed using SPSS version 25.0 software package. Descriptive analysis will be performed to present frequency distribution for demographic and sexual characteristics. Chi-square test will be performed to establish the relationship between independent variables and dependent variable. Thereafter, univariate and multivariable logistic regression will be conducted to determine the motivators for family planning utilization among youth. All independent variable with p-value of less than or equal to 0.2 at binary logistic regression models will be included in multivariable logistic regression model. All independent Variables with p-value of $\leq$ to 0.05 will be regarded as significant.

Dissemination of results

The findings of this study will be disseminated to the following; University of Dodoma, authorities of the respective higher learning institution, Ministry of Health. Furthermore, manuscript will be presented and submitted at a peer reviewed journal for publication and presented at local and international conference.

Ethical approval and consent to participate

This study was submitted to the Directorate of Research, Publication and Consultancy of the University of Dodoma for ethical approval. The ethical committee has assessed and given the ethical approval for this study Ref. No. MA.84/261/02/'A'/91. Furthermore, permission for research conduction will be obtained from Regional Administrative Secretary (RAS) of Dodoma Region. Also, the researcher will present an authorization letter to introduce the researcher to the authority of the respective higher learning institution where the investigator is expecting to conduct the research. An individual informed consent both verbal and written will be obtained from study participant after been full informed about the study goals and processes involved. Participants will be ensured about privacy and confidentiality. Anonymity will be maintained by the use of code number on the questionnaire instead of participant's name and participant will have absolute right and freedom to withdraw from the study at any time.

Discussion

According to national surveys, including the Tanzania Demographic and Health Survey (TDHS) 2015–2016, utilization of family planning services and other SRH education remain limited among the majority of adolescents [8]. About 68% of women of reproductive age (WRA), including adolescents are not using FP services in Tanzania, Dodoma inclusive [19]. Similarly, the rate of teenage pregnancy stands at 27% according to 2015/16 TDHS. By the age of 19 years, almost half (44%) of the women are either mothers or are pregnant with their first child [19]. As a result, many youth continue to engage in risky sexual practices and behaviors such as safe sexual partnerships and inconsistent condom use [8]. These risk behaviors damp them into teenage pregnancies which in turn result in higher risk maternal morbidity and mortality [19]. A recent scoping review reported adverse consequences of teenage pregnancy such as unsafe abortion and maternal deaths in Tanzania [24], and is considered to contribute to the national stand of 556 MMR per 100,000 live births [19], and 512 MMR per 100, 000 per live births in Dodoma region, according to population and Housing Census in 2012. A study conducted in rural Tanzania found that; age, marital status, geography and health system factors; such availability of commodities, understaffing and availability of unskilled health care providers were associated with uptake of family planning [25].

This is not risky

The modern contraceptive use among adolescent accounts for only 40% for unmarried sexually active adolescent in Tanzania [26] while the study conducted in St. John University in Dodoma found that, the prevalence of family plan use was 47% among undergraduate students [21].

Consequences of not utilizing family planning services may results to adolescents not attain their educational goal, teenage pregnancies, unwanted pregnancies which can leads to unsafe abortion and STIs including HIV/AIDs, this may also leads morbidity and mortality and ultimately to over use of government expenditure unnecessarily to health services [4]. In Dodoma teen pregnancy accounts for about 39% which is higher compared with other regions [19].

Therefore, findings from this study expected to bridge knowledge gap by addressing motivators for family planning utilization among higher learning institution youth students in Dodoma Tanzania, which will then help to improve utilization of family planning services.

Limitations

The following are the limitations of this study;

1. This study will be a cross-sectional study hence not useful at establishing the causal effect relationship between family planning utilization and their motivators
2. All assessment will depend on self-reports by the participants with likely to be a cause of informational biases.

Author's contribution

Conceptualization: Upendo Munuo, Fabiola Vincent Moshi

Methodology: Upendo Munuo, Fabiola Vincent Moshi

Supervision: Fabiola Vincent Moshi

Writing- original draft: Upendo Munuo

Writing- review &editing: Fabiola Vincent Moshi

Acknowledgement

Authors would like to appreciate and thanks Ministry of Health for supporting the tuition fees and the fund for conducting this study, the University of Dodoma Research Committee for granting ethical clearance for undertaking this study. Furthermore, the authors would like to acknowledge School of Nursing and Public Health at the University of Dodoma for their continuous support in development of this study protocol.

References

1. World Health Organization. Making health services adolescent friendly. Developing national quality standards for adolescent friendly health services. 2012. p. 56.

2. World Health Organization. Adolescent health. WHO Africa, Health Topics. 2020.

3. Dennis ML, Radovich E, Wong KLM, Owolabi O, Cavallaro FL, Mbizvo MT, et al. Pathways to increased coverage: An analysis of time trends in contraceptive need and use among adolescents and young women in Kenya, Rwanda, Tanzania, and Uganda. Reprod Health. 2017;14:130.

4. Dangat CM, Njau B. Knowledge , attitude and practices on family planning services among adolescents in secondary schools in Hai District , northern Tanzania. 2013;15(1):1–8.

5. International Center for Research on Women (ICRW). Adolescents and family planning: what the evidence shows. J R Coll Gen Pract. 2014;17(81):1–12.

6. Chandra-Mouli V, Camacho AV, Michaud PA. WHO guidelines on preventing early pregnancy and poor reproductive outcomes among adolescents in developing countries. J Adolesc Heal. 2013;52(5):517–22.

7. Radovich E, Dennis ML, Wong KLM, Ali M, Lynch CA, Cleland J, et al. Who meets the c ontraceptive needs of young women in Sub-Saharan Africa? J Adolesc Heal. 2018;62(3):273–80.

8. WHO. Report of an adolescent health services barriers assessment (AHSBA) in the United Republic of Tanzania with a focus on disadvantaged adolescents. 2019. p. 1–2.

9. United Nations. Transforming our world: the 2030 agenda for sustainable development. 2016.

10. Smith J. Improving adolescent access to contraception in Sub-Saharan Africa : A review of the evidence. Afr J Reprod Health. 2020;24(1):152–64.

11. United Nations. Report of the international conference on population and development. Report of the International Conference on Population and Development. A/94/10/18. Programme of Action. 1995. 11–12 p.

12. Godia PM, Olenja JM, Lavussa JA, Quinney D, Hofman JJ, Van Den Broek N. Sexual reproductive health service provision to young people in Kenya; Health service providers' experiences. BMC Health Serv Res. 2013;13(1).

13. Dansereau E, Schaefer A, Hernández B, Nelson J, Palmisano E, Ríos-Zertuche D, et al. Perceptions of and barriers to family planning services in the poorest regions of Chiapas, Mexico: A qualitative study of men, women, and adolescents. Reprod Health. 2017;14(1):1–10.

14. Makola L, Mlangeni L, Mabaso M, Chibi B, Sokhela Z, Silimfe Z, et al. Predictors of contraceptive use among adolescent girls and young women (AGYW) aged 15 to 24 years in South Africa: results from the 2012 national population-based household survey. BMC Womens Health. 2019;19:158.

15. Darroch JE, Woog V, Bankole A. Additing it up: Costs and benefits of meeting the contraceptive needs of adolescents. New York Guttmacher Inst. 2016;(May):1–16.

16. Michalow J, Chola L, McGee S, Tugendhaft A, Pattinson R, Kerber K, et al. Triple return on investment: The cost and impact of 13 interventions that could prevent stillbirths and save the lives of mothers and babies in South Africa. BMC Pregnancy Childbirth. 2015;15:39.

17. National Bureau of Statistics. Tanzania dermographic and health survey 2015-2016. Dar Es Salaam: Ministry of Finance; 2016.

18. NBS. Tanzania in Figures 2018 National Bureau of Statistics Dodoma. United Republic of Tanzania. 2019. 103 p.

19. Tanzania UR of. Tanzania demorgraphic and health survey Indicator Survey (TDHS-MIS) 2015-2016. 2016;

20. Cochran`s. Determination of appropriate Sample Size. 1977;

21. Kara WSK, Benedicto M, Mao J. Knowledge , Attitude , and Practice of Contraception Methods Among Female Undergraduates in Dodoma , Tanzania Socio-demographic characteristics of respondents. 2019;11(4).

22. Casey SE, Gallagher MC, Kakesa J, Kalyanpur A, Muselemu B, Rafanoharana RV, et al. Contraceptive use among adolescent and young women in North and South Kivu , Democratic Republic of the Congo : A cross- sectional population-based survey. PloS Med. 2020;17(3):e1003086.

23. Shah CJ, Solanki V, Mehta H. Attitudes Of Adolescent Girls Towards Contraceptive Methods. Australas Med J. 2011;4(1):43–8.

24. Tull K. Maternal and sexual reproductive health situation in Tanzania. 2020;4–5.

25. Carroll A, Kapilashrami A. Barriers to uptake of reproductive information and contraceptives in rural Tanzania: An intersectionality informed qualitative enquiry. BMJ Open. 2020;10(10):1–8.

26. Ministry of Health, Community Development, Gender E and C. The National Road Map Stratergic Plan To Accelerate Reduction of Materna and New boRN and Child Deaths in Tanzania 2008-2015. PhD Propos. 2015;1.

Ethical clearance

Click here to access/download
Supporting Information
UPENDO.PDF
