# Supplementary material for: Assessing motivators for utilizing family planning services among youth students in higher learning institutions in Dodoma, Tanzania: Protocol for analytical cross sectional study
Source: PLoS One. 2023 Mar 10;18(3):e0282249. doi: 10.1371/journal.pone.0282249 (PMC10004694; doi:10.1371/journal.pone.0282249)
Supplement: Attachment — Submitted filename: Response to Reviewers PLOS ONE.docx [file pone.0282249.s004.docx]

**Response to Reviewers’ comments**

| **Source of comments** | **Comments** | **Response** | **Page and line number** |
| --- | --- | --- | --- |
| Reviewer 1 | - The author describes using an interview administered and self- administered questionnaire. Which method will be used? - The questionnaire is not attached | - Thank you. The comment taken and correction made therefore self-administered questionnaire will be used as a method of data collection. - The questionnaire is now attached (***see additional file)*** | Pg no.2 and line no. 34 |
|  | - The reasons for stratified sampling is not stated. - It is also not stated how the data analysis would be adjusted to accommodate the stratification | Sorry, there is no stratification. Unfortunately, the word stratified in sampling was written by mistake and now it is omitted. | Pg no.8 and line no. 196 |
|  | - What is the existing knowledge gap in this regards in Tanzania or similar countries? - Is there a knowledge gap/ - Is the problem not one of converting existing knowledge into policy and practice? | The knowledge gap is addressed | Pg no.4-5 and line no. 99-121 |
|  | - The protocol is in standard English, however there are numerous typographical and grammatical errors | Thank you for the comment. Typographical and grammatical errors are corrected | Throughout the manuscripts |
| Reviewer 2 | No | Thank you for your highly valuable and appreciated inputs and recommendations. |  |
| Reviewer 3 | Recommendations  **Introduction**   - Include effective family planning methods in other MLIC countries. Provide more details of the family planning methods that exist in Dodoma city. | Thank you for your comment. The information about the available and recommended family planning method for youth is added. | Pg no. 4 and line no. 84 – 92. |
|  | **Methods**   - Provide more information about the institutions where the students are studying - To report if the questionnaire have been tested for internal validity | - Information about the institutions where the students are studying is added - Information about if the will be questionnaire will tested for internal validity is added. | Pg no. 6 and line no. 155 - 164 |
